# SOCS3 mRNA expression and polymorphisms as pretreatment predictor of response to HCV genotype 3a IFN-based treatment

**DOI:** 10.1186/s40064-016-3506-5

**Published:** 2016-10-21

**Authors:** Rabia Aslam, Syed Mohsin Raza, Humeira Naeemi, Bushra Mubarak, Nadeem Afzal, Saba Khaliq

**Affiliations:** 1 Department of Physiology & Cell Biology, University of Health Sciences Lahore, Khayaban-e-Jamia Punjab, Lahore, Pakistan; 2 Allied Health Sciences, University of Health Sciences Lahore, Lahore, Pakistan; 3 Department of Immunology, University of Health Sciences Lahore, Khayaban-e-Jamia Punjab, Lahore, Pakistan

**Keywords:** SOCS3, HCV, Interferon therapy, Responders, Relapsers

## Abstract

**Aim:**

Suppressor of Cytokine Signaling 3 (SOCS3) gene belongs to SOCS family as one of the negative regulators of cytokine signaling and IFN response that function via the JAK-STAT pathway in antiviral response. SOCS3 expression and genetic polymorphism influences the pathogenesis and outcome of antiviral treatment in hepatitis C virus (HCV) infected patients. This study was designed for analysis of SOCS3 gene expression and polymorphism in Pakistani HCV patients.

**Methods:**

This descriptive study was conducted on 250 diagnosed HCV genotype 3a infected subjects. The study population was divided into two major groups on the basis of therapeutic response i.e. sustained virological response (SVR) and non-responders/relapsers (NR). SOCS3 gene mRNA expression analysis was done by using Real time PCR technique, whereas ARMS PCR technique was used for analysis of SOCS3 gene polymorphisms i.e. 8464 A/C (rs12952093), −4874 A/G (rs4969170) and −1383 A/G, (rs4969168).

**Results:**

Gene expression analysis of SOCS3 showed that there was statistically significant increase of 2.275-fold and 3.72-fold in relative gene expression for SVR and NR as compared to normal healthy samples (p < 0.001). The distribution of rs4969168, rs4969170 and rs12952093 genotype frequencies between SVR versus NR group were not statistically significant, only the allelic frequency of rs4969170 was statistically significant (p ≤ 0.0001) with therapeutic response.

**Conclusion:**

The gene expression analysis of SOCS3 showed a clear difference in mRNA expression of SOCS3 as a possible indicator of therapeutic response rather than polymorphism of SOCS3 gene in our studied population.

## Background

Cytokines including interleukins, interferons (IFNs i.e. IFN-α, IFN-β, and IFN-ω) and hemopoietins activate a potent positive feedback mechanism which produces high concentrations of IFNs locally after viral infection. IFNs bind to IFN receptors to activate JAK-STAT signaling pathway which transmits information to cause transcription of genes in response to infection (Fallahi et al. 2012; Stevenson et al. 2013). There are a number of negative regulators i.e. SOCS, USP18, PIAS and TcPTP which control the IFN signaling. Suppressor of cytokine signaling (SOCS) proteins, an important negative regulators of JAK-STAT signaling, consists of eight members SOCS1 to SOCS7 and the cytokine-inducible Src homology 2 domain-containing protein (CIS) (Luedde et al. 2001). Out of these, SOCS3 strongly interacts with activated cytokine receptors such as gp130, to negatively regulate STAT3 phosphorylation via kinase inhibitory region (KIR) in its N-terminal domain (Seki et al. 2008). SOCS3 simultaneously binds to cytokine receptors and JAK1, JAK2, and TYK2 (but not JAK3), and inhibits the catalytic domain of the kinases (Babon et al. 2012).

Hepatitis C virus (HCV), a major cause of acute or chronic liver disease, is a non-cytopathic hepatic virus which interacts with the host immune system in a complex way. During a primary infection, there is a rapid increase in HCV RNA level in first few days and remains high throughout the incubation period i.e. 10–12 weeks (Thimme et al. 2001). Worldwide more than 170 million people are infected with this virus, whereas in Pakistan, approximately 10 million people are infected with HCV (Anwar et al. 2013; Rana et al. 2013). Standard therapy against HCV is a combination of immune modulator plus an antiviral agent [Pegylated interferon α (PEG-IFN-α) and guanosine analog ribavirin], which predicts only a 50 % sustained virologic response (SVR; if HCV RNA remain undetectable at 6 months post treatment completion), depending upon HCV infected genotype (Tsubota et al. 2011). HCV genotype 3, most prevalent type in Pakistan, had been considered a good responsive genotype against standard treatment. But recent studies reported it as a difficult to treat genotype both in traditional and direct acting antivirals (DAA) treatment (Ampuero and Romero-Gomez 2015). Besides being expensive in a country like Pakistan HCV treatment has many potential severe side effects so several attempts are being in process to predict a successful therapeutic outcome.

HCV has been reported to reduce SOCS1 expression and up-regulate SOCS3 expression to facilitate HCV replication (Miyoshi et al. 2005; Collins et al. 2014). Recently, Shao et al. (2010, 2013) reported that overexpression of SOCS3 suppress HCV replication in vitro in an mTOR-dependent manner, and SOCS1 promotes HCV replication by blocking the anti-viral activity of IFN, suggesting that both proteins i.e. SOCS1 and SOCS3 act via alternative mechanisms regarding regulation of IFN activity. HCV core and NS5a proteins interacts with SOCS3 resulting in impaired IFN signaling (Miyoshi et al. 2005). This complex mechanism employed by HCV in relation to SOCS gene might represent a crucial step in HCV infection and pathogenesis, including suppressed inflammation and even hepatocellular cancer progression. In vitro studies have demonstrated that a high level of SOCS3 expression was found in HCV-replicating cells which were resistant to IFN therapy (Persico et al. 2007). Recent in vivo studies have demonstrated a positive correlation between high pretreatment SOCS3 gene expression and nonresponse to therapy in genotype 1-infected patients both in the liver as well as in peripheral blood mononuclear cells (PBMCs) (Walsh et al. 2006; Persico et al. 2008; Kim et al. 2009).

Persico et al. (2008) studied three SOCS3 genetic polymorphisms, 1383 A/G, (rs4969168), −4874 A/G (rs4969170) and −8464 A/C (rs12952093) and reported that these genetic polymorphisms in chronic hepatitis C regulate the expression of SOCS3 and interferon treatment outcome. Among these variants, rs4969168 is known as the transcriptional regulator of the gene whereas information regarding the function other two polymorphisms is not yet defined, but are considered to be linked with SOCs3 expression. Out of these SOCS3 polymorphisms AA genotype of rs4969170 is strongly associated with antiviral therapy in HCV patients that might be associated with high expression of hepatic SOCS3 (Persico et al. 2008; Kim et al. 2009). In a recent report, Angelo et al. (2013) studied that combination of MxA, OPN and SOCS3 protective genotypes was more frequent among the SVR patients. The combined analysis of SOCS3 and IL28B protective genotypes (G/G + C/C) had greater power than IL28B to predict SVR. The G/G genotype was more generally detected in the sustained virological responders, whereas the A/G + A/A genotype was more frequent in NR/R subjects (Angelo et al. 2013). However, Hamdi et al. (2012) reported no significant difference in SOCS3 gene expression between responders and non-responders in HCV-genotype 4 patients. Similar to SOCS3 other genes such as STAT3 are also found to be associated with HCV infection. Expression levels of STAT3 are found to be reduced in HCV-infected patients, Huh-7 cells transfected with HCV constructs and also in HCV-infected liver tissue samples (Stevenson et al. 2013; Li et al. 2012). The expression of STAT3 along with SOCS3 has been recently found to be associated with IFN treatment in HCV infection and HCV-infected cell line (Ryan et al. 2012; Zhao et al. 2016).

To the best of our knowledge it is the first study conducted in Pakistan relating SOCS3 and STAT3 expression and SOCS3 SNP profiling in HCV infected patients aimed with the prediction of therapeutic outcome in HCV patients in our population.

## Methods

### Patients cohort

All (250) HCV infected participants were recruited from outdoor patient (OPD) Department of Gasteroenterology, Jinnah Hospital Lahore and Department of Gastroenterology and Hepatology, Sheikh Zayed Hospital, Lahore during Oct-2013 to April 2015. The study protocol was approved from the Institutional Ethical Review Committee, and Advance Studies and Research Board, University of Health Sciences Lahore. An informed written consent was obtained with all the relevant details such as age, gender, clinical findings, duration of disease and therapeutic history in a structured questionnaire. An approximately, 6 ml of blood sample was collected for RNA and DNA isolation. Healthy volunteers (100) were also recruited in the current study after informed consent and clinical testing of HCV, HBV and any other disease.

The inclusion criteria for the study was as follows: individuals age ≥18 years of either gender, diagnosed HCV genotype 3a patients (Both ELISA and PCR positive) with Peg IFN plus ribavirin treatment and on relapse of HCV infection after the completion of therapy were included. Patients were excluded if they were positive for HBV or any other known viral infection, any type of liver diseases, malignancy i.e. HCC and others. These patients were divided into two groups depending upon the treatment response i.e. Sustained virological response (SVR) and non-responders/relapse (NR). SVR: is defined as an undetectable serum HCV after 6 months of combination therapy. NR: is defined as a detectable serum HCV level after 6 month of combination therapy or a virological relapse occurs when HCV RNA reappears in a patient who had completed the treatment with negative HCV RNA as an indicator of end-of-treatment response.

### SOCS3 and STAT3 gene expression analysis

To evaluate the effect of SOCS3 and STAT3 gene expression in HCV infected patients with different therapeutic response, total RNA was extracted from the blood sample of HCV patients and normal (healthy) volunteers followed by gene expression analysis. Due to problems in sample collection from all the patients at different time intervals, the RNA used for the Real Time PCR analysis consists of samples taken before start of therapy (later on designated as SVR or NR group) and from patients who already had a relapse of disease after completion of combination therapy. Briefly, mRNA expression of SOCS3 and STAT3 gene was carried out after RNA extraction from freshly collected blood samples after isolating peripheral blood mononuclear cells (PBMCs). Total cellular RNA was extracted from PBMCs using the TriZol reagent (Invitrogen, USA) following the manufacturer’s instructions. Total cellular RNA (1 μg) was reverse transcribed into single-stranded complementary DNA (cDNA) using first strand cDNA synthesis kit (Thermo scientific, USA). The mRNA expression level of SOCS3 and STAT3 gene as well as GAPDH, a housekeeping gene used as internal control for normalization (Primer sequences in Table 1), was detected on iQ5 icycler Real Time PCR detection system (Biorad, USA). Each reaction on Real Time PCR was performed in triplicate. The relative gene expression analysis was done by using iQ5 2.1 software. Results obtained were analyzed statistically for relative gene expression changes by student’s *t* test and one-way ANOVA where p < 0.05 was considered as statistically significant.

**Table 1:** Sequences of primers for PCR amplification

No	Gene	Primer	Product size
1.	rs12952093-1	AACACTTGTTTTTTGTTTGACACAGTACCC	Common amplicon = 449 bp C allele = 204 bp A allele = 294 bp
	rs12952093-2	CACTCCAGCCTGGGCAACAAAT	
	rs12952093-3	CTCCCCCGATAATTGCAAACAAAAT	
	rs12952093-4	AACATGGCAAACACCGTCTCTACCT	
2.	rs4969170-1	TCTTTCCATTGTTTTTAGAGACCCCA	Common amplicon = 375 bp G allele = 235 bp A allele = 196 bp
	rs4969170-2	AACCAAAAAGTACTCTAGAAGAAAGCATGC	
	rs4969170-3	AAAGACGGAAAAAGGCAGACACTCT	
	rs4969170-4	CCACATTTTCAGAAACGTTTTCGTC	
3.	rs4969168-1	AGACCAGCTGACCAGCCCATACA	Common amplicon = 211 bp A allele = 120 bp G allele = 138 bp
	rs4969168-2	GGGGAAGCAACATTTGGAGGGTAC	
	rs4969168-3	ACCAGGAGCCTGAGGTGAAAGATGT	
	rs4969168-4	GACAGTCACCGAAAACACAGGTTCC	
4.	SOCS3-F	GGAGACTTCGATTCGGGACC	131 bp
	SOCS3-R	GAAACTTGCTGTGGGTGACC	
5.	STAT3-F	GGCATTCGGAAAGTATTGTCG	265 bp
	STAT3-R	GGTAGGCGCCTCAGTCGTATC	
6.	GAPDH-F	ACCACAGTCCATGCCATCAC	453 bp
	GAPDH-R	TCCACCACCCTGTTGCTGTA	

### Genotyping of SOCS3 gene

Three SOCS3 gene polymorphisms i.e. −8464 A/C (rs12952093), −4874 A/G (rs4969170) and 1383 A/G (rs4969168) were chosen for genotyping. Genomic DNA was extracted from PBMCs of samples using standard Phenol–Chloroform method. The extracted DNA was subjected to genotyping using amplification refractory mutation system-polymerase chain reaction (ARMS-PCR) of SOCS3 specific regions spanning the polymorphic sites. For each assessed polymorphism, the assay included tetra-primer pair (Table 1). ARMS-PCR amplifications were carried out in 15 µl reaction mixtures consisting of 1 µl of diluted DNA (50 ng/µl), 10 × PCR buffer, 2.5 mM MgCl_2_, 0.4 µl of mixture containing 100 µM of each dNTP, 0.2 mM of inner primers, 0.4 mM of outer primer and 1U (unit) of *Taq* DNA polymerase (Thermo scientific, USA). Temperature profile consisted of an initial denaturation at 94 °C for 4 min followed by 35 cycles of denaturation at 94 °C for 30 s, annealing at 62 (rs12952093 and rs4969170) and 64 °C (rs4969168) for 30 s and extension at 72 °C for 40 s. and a final extension step at 72 °C for 5 min.

### Statistical analysis

Genotype and allele frequency for the SNPs was calculated by the Online Genetic Epidemiology tool OEGE (http://www.oege.org). Same software was used to asses Hardy–Weinberg equilibrium using the Pearson goodness-of-fit *χ* ^2^ test with 1° of freedom for biallelic markers. For other variables mean ± SD was calculated for quantitative variables. On the contrary, qualitative variables were expressed as frequencies and percentages. Chi square test was applied to measure the association of general characteristics among the study groups while One-way ANOVA was used to compare the mean of quantitative parameters among the study group. In order to evaluate the association of polymorphism with the study groups’ four genetic models (Co-dominant Model, Dominant Model, Recessive Model and Allele Model) were constructed. Crude and adjusted Odds ratios (ORs) with their 95 % confidence intervals (CI) of the association of polymorphism with the study groups was estimated by the logistic regression. For adjusted ORs and 95 % CI adjustment was made by potential confounders such as age, gender and viral titer. All the data analysis was performed by IBM SPSS ver. 21 while p value <0.05 was considered as statistically significant.

## Results

### Patient characteristics

**Fig. 1:**
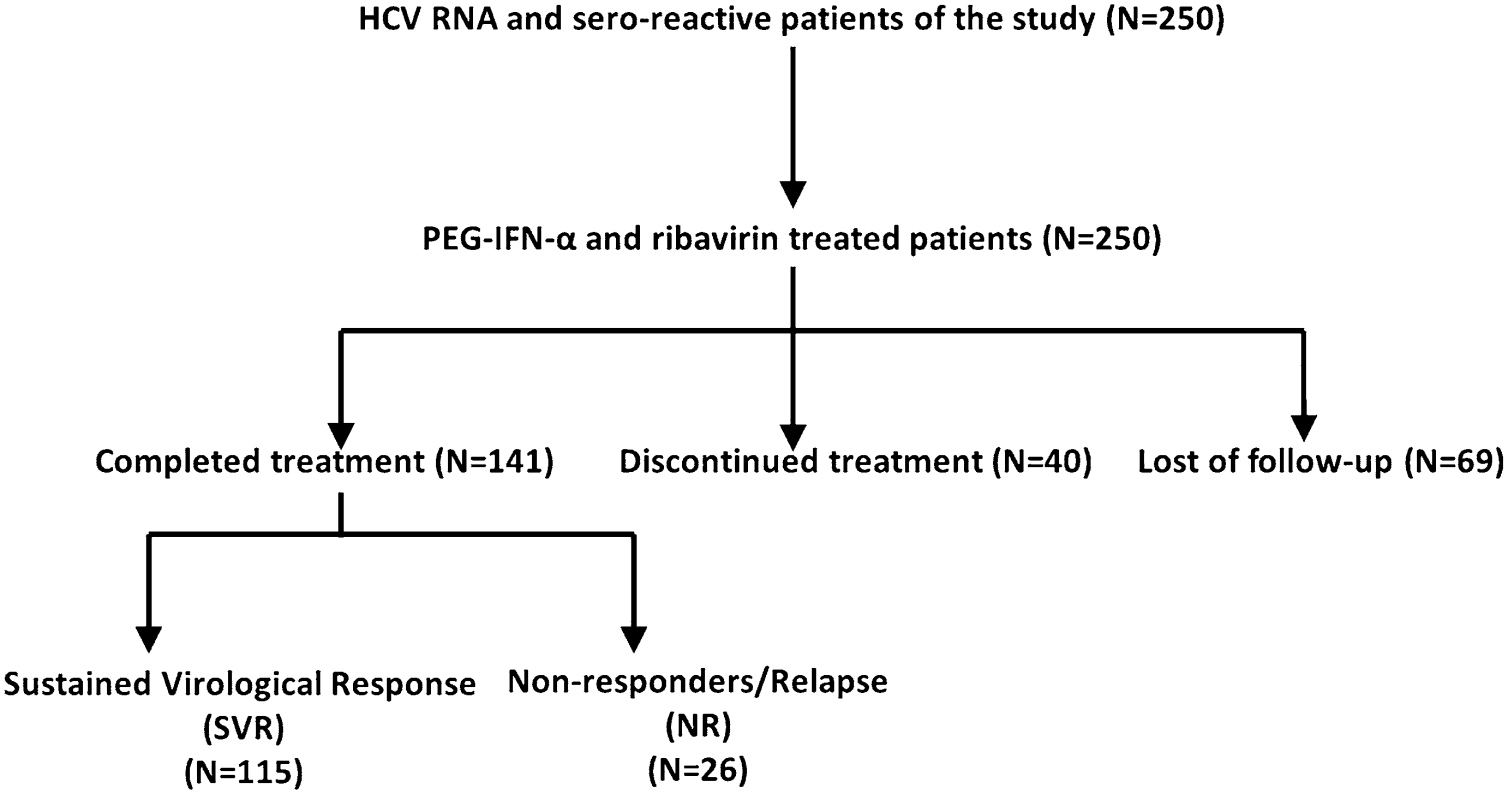
Flow chart depicting the sample size of HCV patients enrolled in the study and response rate to treatment

It was a descriptive study with follow-up to determine the response of the subject against PEG-IFNα plus ribavirin therapy, periodic follow-up of each patient was made till June, 2015. Out of total 250 participants, 69 patients discontinued the therapy because of side effects and financial constraints, whereas 40 patients were lost to follow-up (Fig. 1). A total of 141 eligible patients who completed the Peg-IFN-α plus ribavirin therapy, with viral titer of ~10^4^–10^7^ IU/ml at the time of diagnosis were screened for further analysis. After completion of treatment 115 patients achieved SVR (82.6 %) and 26 patients either were non-responders or had a relapse of disease. HCV infected patients with SVR and NR were compared for gender, age, ALT, AST and viral titre. The categorization of male patients on the therapeutic response showed that 77.6 % achieved SVR, whereas 87.5 % females showed SVR. Almost a double number of males (22.4 % out of total male patients) showed a relapse after treatment as compared to female patients (12.5 % out of total male patients) but overall the difference of gender was not statistically significant with therapeutic response (p = 0.37). The overall mean age in all patients was 40.79 ± 9.8 while relapse patients had high mean age 43.58 ± 11.6 with p value 0.0344. In case of ALT and AST overall mean ± SD was 92.22 ± 71.5 and 104.96 ± 82.9, NR had highest mean ± SD of ALT and AST i.e. 203.27 ± 7.2 and 229.31 ± 92.1 than SVR (p = 0.0001). Similarly, bilirubin concentration was significantly associated in SVR and NR group i.e. 23.2 ± 7.32 and 27.2 ± 10.98 with p = 0.024. HOMA-IR of the HCV patients was also calculated, but no significant difference was observed between both groups (p = 0.45; Table 2).

**Table 2:** Demographic and clinical characteristics of HCV infected patients with different therapeutic response

	Overall mean/median	SVR mean/median	Non-responders/relapser mean/median	p value
Gender (n, %)				
Male	85 (60.2 %)	66 (77.6 %)	19 (22.3 %)	0.317
Female	56 (39.7 %)	49 (87.5 %)	7 (12.5 %)	
Body mass index (kg/m^2^)	22.43 ± 4.3	22.31 ± 3.5	22.98 ± 7.2	0.48
Age (years)	40.0 ± 9.5	39.2 ± 8.9	43.58 ± 11.6	0.0344
ALT (IU/ml)	90.9 ± 65.5	64.5 ± 41	203.27 ± 7.2	0.0001
AST (IU/ml)	102.34 ± 84.1	73.64 ± 48.3	229.31 ± 92.1	0.0001
Albumin (g/l)	40.95 ± 10.56	41.23 ± 10.98	39.76 ± 8.54	0.52
Total Bilirubin (mg/dl)	23.93 ± 8.22	23.2 ± 7.32	27.2 ± 10.98	0.024
Fasting serum glucose (mg/l)^a^	95 (82–99)	94 (83–99)	96 (82–99)	0.84
Fasting insulin (μU/ml)^a^	6.5 (3–9)	6 (3–9)	7 (4–4.7)	0.43
HOMA-IR^a^	1.29 (0.74–3.25)	1.12 (0.74–2.11)	1.46 (0.77–3.25)	0.45
Viral titer (IU/ml)				
<100,000	68	56	12	0.706
>100,000	73	59	14	

* *p* ≤ 0.05 statistically significant

^a^Data only available for 87 patients

### Expression analysis of SOCS3 and STAT3 gene

**Fig. 2:**
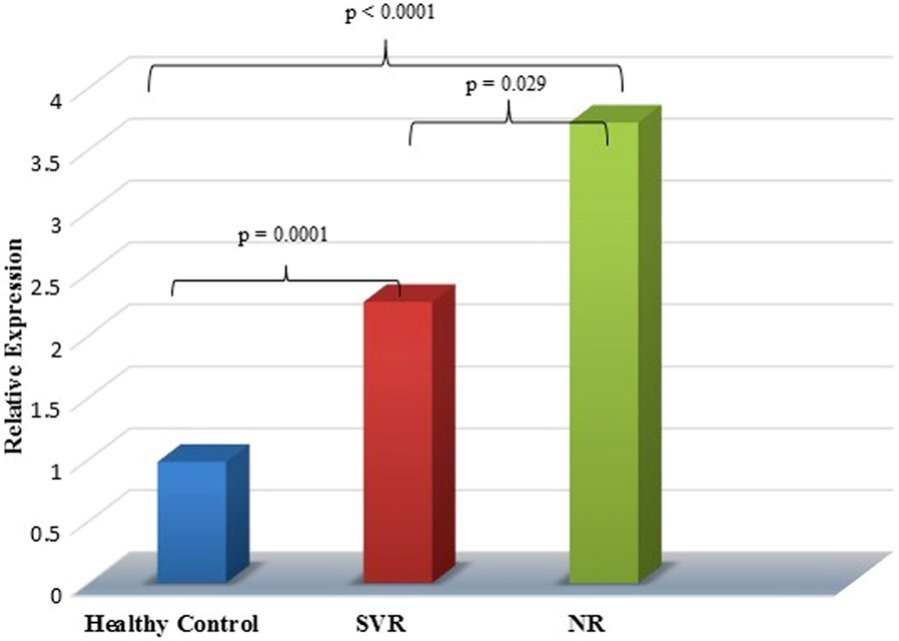
SOCS3 gene expression in PBMCs from healthy volunteers and HCV patients (SVR and NR group) prior to the initiation of PEG-IFNα and ribavirin treatment. p value ≤0.05 was considered as statistically significant

To evaluate the effect of SOCS3 gene expression, known to suppress antiviral activity via the IFN signaling pathway, in HCV infected patients with different therapeutic response, total RNA was extracted from the blood of HCV patients and normal (healthy) volunteers followed by gene expression analysis. Considering the baseline expression level of SOCS3 in healthy controls as 1.0, a statistically significant (p < 0.001) up-regulation of relative gene expression of 2.27 ± 1.18 and 3.72 ± 1.08 folds was observed in SVR and NR group of patients respectively. Moreover, the difference between healthy controls versus SVR, healthy control versus NR and SVR versus NR was also found to be statistically significant i.e. p = 0.0021, p = 0.0001, and p = 0.0029 respectively. Overall, the difference in SOCS3 gene expression in this study was found to be associated with the response to combination therapy (p < 0.0001; Fig. 2).

**Fig. 3:**
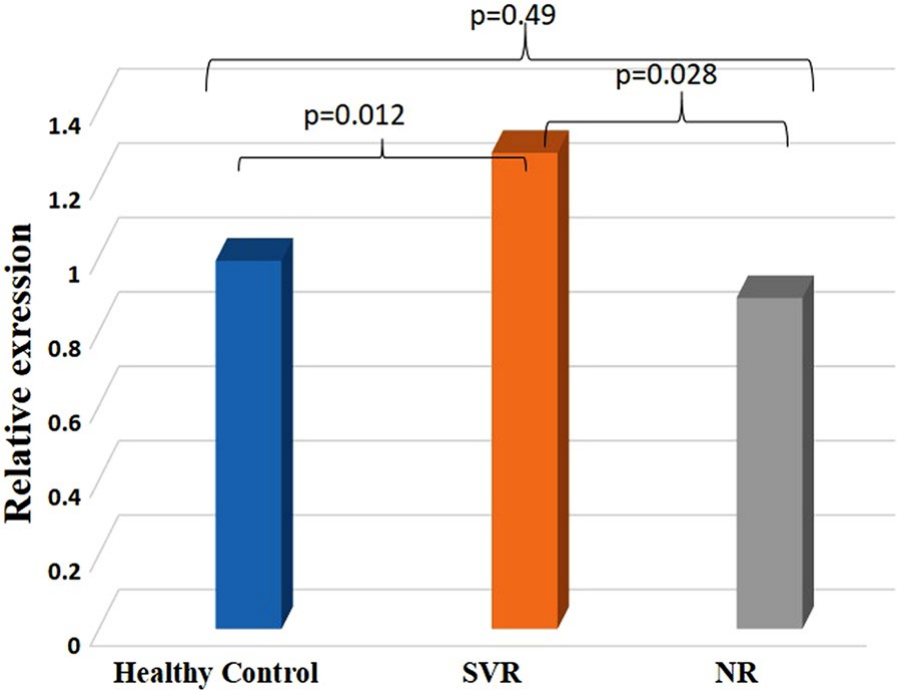
STAT3 gene expression in PBMCs from healthy volunteers and HCV patients (SVR and NR group) prior to the initiation of PEG-IFNα and ribavirin treatment. p value ≤0.05 was considered as statistically significant

Similarly, STAT3 gene expression was studied in HCV patients and normal (healthy) volunteers. Considering the baseline expression level of STAT3 in healthy controls as 1.0, a statistically significant (p = 0.012) up-regulation of relative gene expression of 1.28 ± 0.29 was observed in SVR group, whereas down-regulation of expression i.e. 0.89 ± 0.43 folds was observed in NR group of patients (p = 0.49) in comparison with healthy controls. Moreover, the difference of expression levels between SVR versus NR was also found to be statistically significant i.e. p = 0.028 (Fig. 3).

### Genotypes frequencies of SOCs3 polymorphism and their association with SVR and NR in HCV patients

The population was tested for HWE for SOCs3 polymorphisms, healthy volunteers were in HWE for all three polymorphisms under study. SOCS3 polymorphism rs4969170 and rs12952093 were in HWE among the individuals belonging to SVR group (p < 0.0001, but only rs12952093 was in HWE among the individuals of NR group (p < 0.0001). SOCS3 polymorphism rs4969168 was not in HWE among both groups i.e. SVR and NR. Linkage disequilibrium analysis showed that all SNPs under study were weekly associated with HCV infected groups.

 In order to evaluate the association of polymorphisms rs4969168, rs4969170 and rs12952093 with study population, three genetic models (co-dominant, recessive, and dominant) were constructed with crude and adjusted ORs with 95 % CI. After the statistical analysis the results of all three SNPs i.e. the genotype distributions and association between overall treatment responses are shown in Table 3. There were no significant difference in the genotype or allele distribution of rs4969168 G/A and rs12952093 C/A in any studied model with p > 0.05 before and after adjusted OR. Distribution of rs4969170 A/G genotype also showed no significant association with response in both groups, whereas the allele model showed that about 28.8 % of the patients with NR had effecting allele while in SVR its frequency was 64.3 % which was statistically significant p ≤ 0.0001 with much greater OR as compared to others (p < 0.0001).

**Table 3:** Distribution of alleles and genotype frequencies and comparison in HCV patients

		Non-relapsers *n* (%)	Relapser *n* (%)	OR crude (95 % CI)	p value	OR Adjusted* (95 % CI)	p value adjusted*
*SOCS3 rs4969168 (G/A) model*							
	Genotype						
Co-dominant	GG	61 (53.0)	12 (46.2)	–	0.759	–	–
	GA	44 (38.3)	12 (46.2)	1.386 (0.570–3.373)	0.471	1.242 (0.498–3.100)	0.642
	AA	10 (8.7)	2 (7.7)	1.017 (0.197–5.238)	0.984	0.872 (0.163–4.658)	0.873
Recessive	GG and GA	105 (91.3)	24 (92.3)	0.875 (0.180–4.255)	0.868	0.784 (0.156–3.928)	0.767
	AA	10 (8.7)	2 (7.7)				
Dominant	GG	61 (53.0)	12 (50.0)	1.318 (0.561–3.095)	0.868	1.173 (0.487–2.828)	0.722
	GA and AA	54 (47.0)	14 (50.0)				
	Alleles						
	G	166 (72.7)	36 (69.2)	0.8675 (0.450 –1.671)	0.6709	0.8675 (0.4503–1.6712)	0.6709
	A	64 (27.8)	16 (30.7)				
*SOCS3 rs4969170(A/G) model*							
	Genotype						
Co-dominant	AA	25 (21.7)	3 (11.5)	–	0.473	–	–
	AG	32 (27.8)	9 (34.6)	2.344 (0.574–9.576)	0.236	1.951 (0.456–8.346)	0.367
	GG	58 (50.4)	14 (53.8)	2.011 (0.531–7.622)	0.304	1.698 (0.427–6.756)	0.452
Recessive	AA and AG	57 (49.6)	12 (46.2)	2.130 (0.591–7.676)	0.753	1.069 (0.445–2.568)	0.881
	GG	58 (50.4)	14 (53.8)				
Dominant	AA	25 (21.7)	3 (11.5)	1.147 (0.488–2.691)	0.239	1.790 (0.473–6.768)	0.391
	AG and GG	90 (78.3)	23 (88.5)				
	Alleles						
	A	82 (35.6)	37 (71.1)	4.452 (2.306 –8.595)	<0.0001	4.452 (2.3060–8.595)	<0.0001
	G	148 (64.3)	15 (28.8)				
*SOCS3 rs12952093(C/A) model*							
	Genotype						
Co-dominant	CC	22 (19.1)	4 (15.4)	–	0.905	–	–
	CA	25 (21.7)	6 (23.1)	1.320 (0.329–5.293)	0.695	1.564 (0.377–6.489)	0.538
	AA	68 (59.1)	16 (61.5)	1.294 (0.391 –4.282)	0.673	1.374 (0.401–4.705)	0.613
Recessive	CC and CA	47 (40.9)	10 (38.5)	1.106 (0.462–2.648)	0.821	1.076 (0.433–2.673)	0.875
	AA	68 (59.1)	16 (61.5)				
Dominant	CC	22 (19.1)	4 (15.4)	1.301 (0.407–4.160)	0.656	1.425 (0.433–4.686)	0.560
	CA and AA	93 (80.9)	22 (84.6)				
	Alleles						
	C	69 (30.0)	14 (26.9)	0.859 (0.437–1.687)	0.660	0.859 (0.437–1.687)	0.660
	A	161 (70.0)	38 (73.0)				

*OR* odd ratio, *CI* confidence interval, *NA* not applicable

* Adjusted on the basis of age, gender, and viral titer. p ≤ 0.05 statistically significant

## Discussion

A major goal of medical genetics during the last two decades has been to unravel the influence of host’s genetics on susceptibility or pathogenesis of common diseases and to find association of human genetic variations with these diseases. Variation in genes which are especially associated with the antiviral pathways influence the response to viral attack, antiviral agents and ultimately the outcome of diseases. Gossens and Nagro (2014) reported that in genotype 3 patients the predictive factors/markers for relapse include male gender (16 vs. 7 %), old age >55 years (27 vs. 12 %), high viral load (20 vs. 7 %) and advanced fibrosis stage (20 vs. 6 %). Recently, Aziz et al. (2016) in a multivariate logistic regression analysis on patients receiving combination therapy from Pakistan reported that 74.8 % patients showed SVR whereas 25.2 % patients were virologicaly non-responders, showing detectable HCV RNA at the end of treatment. The data regarding non-response/relapse to combination therapy in Aziz et al. (2016) study is similar to the results of current study where the relapse rate is 18.4 % and almost a double number of males (22.4 % out of total male patients) showed a relapse after treatment as compared to female patients (12.5 % out of total male patients) but overall the difference of gender was not statistically significant with therapeutic response (p = 0.37) in the current study.

Changes in immune system occurs with increase in age that leads to poor responsiveness to new antigen due to decrease of naïve T cell, less capacity to produce interleukin, changes in cytokine profile, deficiency in T-cell receptor signal transduction and activation (Pawelec et al. 2001; Timm and Thoman 1999). The mean age in SVR and relapse patients in the current study were 39.2 ± 8.96 and 43.58 ± 11.6 indicating a significant association with treatment response (p = 0.0344). Aziz et al. (2016) studied correlation between pretreatment viral load, ALT, AST and SVR and observed HCV RNA level (p < 0.0001), ALT level at the time of treatment (p > 0.003), and steatosis (Non-Fatty liver, p < 0.005) and found a significant association with treatment success. Similarly, in the current study the levels of AST and ALT at the start of treatment were high in NR group (p = 0.0001), but no significant difference in viral load (p = 0.706) was observed.

Recent in vivo studies have demonstrated a positive correlation between high pretreatment SOCS3 gene expression and nonresponse to therapy in genotype-1 infected patients both in the liver as well as in PBMCs (Walsh et al. 2006; Persico et al. 2008; Kim et al. 2009). In the present study, the overall pretreatment SOCS3 mRNA level was higher in patients than in healthy controls (p < 0.001) and this could be explained by the previously reported role of SOCS3 in the negative regulation of the JAK-STAT pathway (Persico et al. 2007, 2008; Kim et al. 2009; Krebs and Hilton 2001). In the present study, a statistically significant increase in pretreatment expression of SOCS3 was observed in SVR versus NR (p = 0.002). The increase in pretreatment SOCS3 mRNA level in HCV genotype 3 infected patients (SVR and NR) as compared to healthy control is in support of the hypothesis that the increase in SOCS3 and resistance to therapy could be HCV genotype dependent. This observation is also in agreement with the recent findings that SOCS3 expression level induced by HCV infection is higher in genotype 1 as compared to genotype 2 (Persico et al. 2007; Kim et al. 2009).

The results of current study are in accordance with the earlier studies on genotype 1 (Persico et al. 2007, 2008; Walsh et al. 2006; Kim et al. 2009) but are in concordance with the study of Jablonowska et al. (2014) who observed higher pretreatment hepatic SOCS3 mRNA expression in responders than in non-responders in genotype 4 patients. Similarly, Pascarella et al. (2013) observed no significant association of expression of SOCS genes (SOCS1, 3 and 7) and prediction of virological response in genotype 3 patients. Jablonowska et al. (2014) suggested that this discrepancy in results could be explained by a genetic variability in SOCS3 since a genetic polymorphism in the SOCS3 gene was reported to influence the level of SOCS3 expression and IFN treatment outcome in chronic hepatitis C (Persico et al. 2008). Persico et al. (2008) studied three SOCS3 genetic polymorphisms, −8464 A/C (rs12952093), −4874 A/G (rs4969170) and 1383 A/G (rs4969168), and reported significant differences in their effects on outcome of antiviral therapy. Particularly, SOCS3 (−4874 AA) genotypes express SOCS3 at elevated levels and consequently have a poorer response to therapy (Persico et al. 2007, 2008; Funaoka et al. 2011). Based on recent findings, the present study aimed to investigate the role of SOCS3 gene polymorphism in resistance to therapy in HCV genotype 3 infected patients since no data concerning this genotype have been reported yet.

In the current study rs4969168 genotypes (GG, GA and AA) and allele frequency (G/A) when compared in SVR versus NR the results were not statistically significant in any model (p = 0.647, p = 0.767 and p = 0.722; Table 3). These findings are in agreement with Persico et al. (2008) who studied that the rs4969168 genotypes were not significantly associated with antiviral therapy where GG genotype was most frequent in both groups with 64.2 % patients in NR and 68.5 % patients in SVR group. In current study, these findings can also be explained by the lack of HWE in the sample population.

Smilarly, rs4969170 genotype (AA, AG, GG) and allele frequency (G, A) distribution was not statistically different between SVR versus NR group of patients (p = 0.367, p = 0.881, p = 0.391 for genotypes and p = 0.67 for allele; Table 3). The results are in concordance with Persico et al. (2008) and Angelo et al. (2013) who studied that rs4969170 AA genotype is strongly associated with failure of the antiviral IFNα therapy in HCV genotype 1 infected patients, the reason of this concordance with current study could be due to the difference in HCV infected genotype i.e. HCV genotype 3a which is now considered as a difficult to treat genotype. However, in the current study a statistically significant association of rs4969170 (A/G) allele frequency with therapeutic response was observed in SVR vs NR groups (p ≤ 0.0001). Angelo et al. 2013 studied that rs4969170 GG genotype frequency was much higher in the SVR patients than in the non-responders/relapse patients.

SOCS3 gene SNP rs12952093 genotypes (CC, CA, and AA) and allele (C/A) frequency distribution was found to have no statistically significant association with therapeutic response when compared between non-relapse vs relapse (p = 0.538, p = 1.076, p = 1.425 of genotypes and p = 0.66 of allele; Table 3). This finding is in concordance with the study of Persico et al. (2008) who studied rs12952093 polymorphism in which 34 % NR and 51.1 % SVR patients showed CC genotype, 48.8 % NR and 40.2 % SVR with CA genotype and 17.3 % NR and 8.7 % SVR with AA genotype with (p = 0.006).

Recent study by Zhao et al. (2016), demonstrated a link between SOCS3, STAT3 and IFN-α signaling triggered by HCV infection and also reporting that STAT3 regulation correlates inversely with SOCS3 induction by IFN-α, which may be important in better understanding the complex interplay between IFN-α and signal molecules during HCV infection. As SOCS3 expression is regulated by other cell-signaling pathways, in the current study we also studied the suggested link of STAT3 and SOCS3 expression by Zhao et al. (2016), with reference to IFN based therapy in our studied population. The expression of STAT3 was down-regulated in NR group (0.89 ± 0.43) which is inversely correlated with the expression of SOCS3, whereas there was a slight increase in expression of STAT3 in SVR group of patients (1.28 ± 0.29). The results of STAT3 down-regulated expression are in agreement with studies on HCV infected patients (Stevenson et al. 2013; Li et al. 2012; Ryan et al. 2012). The expression of STAT3 was not affected by the SOCS3 polymorphisms, similar to the SOCS3 gene expression.

 It is noteworthy that in present study we were not able to establish a link between the.up/down-regulation of SOCS3 and STAT3 expression with SOCS3 SNPs to predict SVR to a combination of PEG-IFNα and ribavirin in chronic hepatitis C genotype 3a infected patients due to overall non-significant association of these SNPs in HCV patients. The down-regulated expression observed in STAT3 gene could be better explained if the full signaling pathways is studied. The molecular mechanisms underlying these associations, as well as their diagnostic and prognostic significance, are worth further studies. To conclude the results, it can be said that genetic factors i.e. polymorphisms alone are not involved in response to therapy in our local population. But the current study has its limitation due to small sample size and collection of samples at different time intervals for the analysis of SOCS3 and STAT3 gene expression.
